# Neutrophil to Lymphocyte Ratio a Prognostic Tool in Endometrial Cancer Among Classical Prognostic Factors

**DOI:** 10.3390/diagnostics15172172

**Published:** 2025-08-27

**Authors:** Alexandra Timea Kirsch-Mangu, Alexandru Țîpcu, Vlad Alexandru Gâta, Diana Cristina Pop, Zsolt Fekete, Alexandru Irimie, Paul Milan Kubelac

**Affiliations:** 1 Department of Oncology, “Iuliu Hatieganu” University of Medicine and Pharmacy, 400347 Cluj-Napoca, Romania; timeakirsch@gmail.com (A.T.K.-M.); alexandru.tipcu@elearn.umfcluj.ro (A.Ț.); drfekete@gmail.com (Z.F.); 2 Prof. Dr. I. Chiricută” Institute of Oncology, 400015 Cluj-Napoca, Romania; gata.vlad@umfcluj.ro (V.A.G.); patcasdiana@yahoo.com (D.C.P.); airimie@umfcluj.ro (A.I.); 3 Department of Surgical Oncology and Gynecologic Oncology, “Iuliu Hațieganu” University of Medicine and Pharmacy, 400012 Cluj-Napoca, Romania; 4 Institute for Advanced Study of Science and Technology, Babeș-Bolyai University, 1 Mihail Kogălniceanu Street, 400347 Cluj-Napoca, Romania

**Keywords:** Endometrial cancer, neutrophil-to-lymphocyte ratio, NLR, prognostic biomarker, systemic inflammation, survival, gynecologic oncology

## Abstract

**Background:** Endometrial cancer (EC) is the most common gynecologic malignancy in developed countries. Despite advances in diagnosis and treatment, recurrence and mortality remain significant concerns. The neutrophil-to-lymphocyte ratio (NLR), a marker of systemic inflammation, has shown prognostic value in several malignancies, but its utility in EC remains underexplored. **Objective:** To evaluate the prognostic significance of the preoperative NLR in patients with endometrial cancer undergoing primary surgical treatment. Methods: We conducted a retrospective cohort study including 398 patients with histologically confirmed endometrial adenocarcinoma surgically treated at a tertiary cancer center. Preoperative complete blood counts were used to calculate NLR, and a cutoff value of 2.27 was determined through Receiver Operating Characteristic (ROC) analysis. Survival outcomes were assessed using Kaplan–Meier analysis and Cox proportional hazards modeling. **Results:** Patients with NLR ≥ 2.27 had significantly reduced median overall survival (OS) compared to those with NLR < 2.27 (72.3 vs. 92.8 months, *p* = 0.008). In multivariate analysis, elevated NLR remained an independent predictor of poorer OS (HR = 1.87; 95% CI: 1.156–3.017; *p* = 0.011), alongside age ≥ 64 years, lymphovascular space invasion (LVSI), lymph node involvement, and distant metastases. ROC analysis yielded an Area Under the Curve (AUC) of 0.646 for NLR. Notably, vaginal brachytherapy was associated with improved survival (HR = 0.53; *p* = 0.026), while other adjuvant therapies were not independently significant. **Conclusions:** Preoperative NLR is an accessible, independent prognostic biomarker in endometrial cancer and may serve as a surrogate indicator of tumor-promoting inflammation and immune dysregulation. Its integration into preoperative assessment could enhance risk stratification and guide personalized treatment strategies. However, findings should be interpreted in light of the study’s retrospective design, single-center setting, and lack of molecular classification data. Prospective validation is warranted to confirm its clinical utility.

## 1. Introduction

Endometrial cancer (EC) is the most prevalent gynecological malignancy in developed countries, with increasing incidence rates observed globally [1]. There have been significant strides in improving diagnostic criteria and treatment options for EC over the past few decades, but recurrence remains a major concern as curing EC can be challenging. Prognostic indicators for early detection of potential recurrence can facilitate timely and optimal treatment, thereby enhancing survival rates.

Identifying reliable prognostic biomarkers is crucial for patient stratification and optimizing treatment protocols. One emerging biomarker is the lymphocyte to neutrophil ratio (NLR), which is indicative of systemic inflammation and has shown promise as a prognostic factor in various cancers. The NLR has been identified as a potential biomarker for cancer prognosis, garnering clinical interest due to its accessibility and the straightforward calculation of the ratio from routine blood cell counts in patients.

Walsh et al. were the first to implement the parameter for prognostic evaluation of cancer patients undergoing colorectal surgery. The results of the retrospective study indicate that NLR serves as a valid immune-inflammatory parameter, predicting the clinical outcomes of cancer patients and demonstrating significant prognostic value, but few studies have evaluated this biomarker as a prognostic factor in EC [2].The strong link between inflammation and cancer progression suggests the significance of increased tumour-associated neutrophils (TAN), which are neutrophils that infiltrate tumours may serve as a prognostic biomarker.

Given the strong association between inflammation and tumors, tumor-associated inflammatory indicators have increasingly emerged as a focal point of research in recent years. NLR serves as superior indicators of systemic inflammation. Inflammation can facilitate tumor angiogenesis, enhance tumor cell proliferation, and suppress tumor cell death, hence establishing a close association between tumors and inflammatory responses; furthermore, certain inflammatory response markers correlate with tumor prognosis. Neutrophils are often associated with tumor promotion, while lymphocytes are involved in anti-tumor immunity [3]. The balance between these cell types, reflected in the NLR, may provide insights into the immune landscape of tumors [4].

Numerous studies have reported on the prognostic significance of the NLR in various malignancies. For example, a meta-analysis by Templeton et al. [5] found that a low NLR was associated with poorer survival outcomes in cancer patients. Research indicates that NLR is significant in the diagnosis, prognosis, and recurrence of tumors [6,7,8,9]. This suggests that NLR could serve as a valuable adjunct in assessing prognosis in EC patients. Conventionally acknowledged independent prognostic markers encompass age, Federation of Gynecology and Obstetrics (FIGO) stage [10], histological grade, histopathological subtype, tumor size, and lymphovascular invasion (LVSI). These characteristics have been extensively utilized in risk assessment and the customization of treatment methods, resulting in a substantial enhancement of prognosis in EC. Nonetheless, these tumor-associated risk factors lack sufficient accuracy in predicting the recurrence risk and prognosis of EC.

In recent years, several studies have specifically examined NLR in the context of endometrial cancer. There are several reports supporting the prognostic significance of systemic inflammatory markers in endometrial cancer. Cummings et al. conducted a comprehensive analysis of 605 surgical patients and demonstrated that elevated preoperative neutrophil-to-lymphocyte ratio (NLR ≥ 2.4) and platelet-to-lymphocyte ratio (PLR ≥ 240) independently predicted both overall and cancer-specific survival [11]. Moreover, patients with simultaneous elevations in both markers had markedly poorer 5-year survival outcomes, underscoring their combined prognostic value. These findings align with the observations of Ural et al., who found significantly elevated NLR levels in endometrial cancer patients compared to those with benign or hyperplastic endometrial tissue, suggesting a consistent role for systemic inflammation in disease progression [12], yet results across studies remain inconsistent and limited by retrospective design and heterogeneous patient populations.

A growing body of evidence substantiates the involvement of inflammation and immunology in carcinogenesis, tumor progression, and prognosis [13]. Peripheral blood cells—such as neutrophils and lymphocytes—serve not only as indicators of systemic inflammation but also reflect the state of tumor-host immune dynamics. Recent work by Sayed demonstrated that basic hematological parameters derived from the complete blood count (CBC), including the neutrophil-to-lymphocyte ratio (NLR), possess significant diagnostic and prognostic value even outside oncology, such as in COVID-19, where they correlated with disease severity and outcome [14]. This underscores the translational relevance of inflammation-based indices like NLR, supporting their broader application as accessible and cost-effective biomarkers across diverse disease contexts, including cancer.

Combining neutrophil and lymphocyte counts, NLR is a measure of systemic inflammation and is thought to be a balanced predictor of both the anti-tumor inflammatory response and the tumorigenic inflammatory response. The elevation of NLR in patients indicates a relative rise in neutrophils and a decline in lymphocytes, hence facilitating the progression of inflammation towards tumorigenic inflammation. Besides the release of vascular endothelial growth factor (VEGF) by neutrophils, VEGF overexpression can facilitate tumor angiogenesis and distant metastasis. Furthermore, the carcinogen released by neutrophils can induce the secretion of tumor tissue and enhance its invasiveness. An elevation in neutrophils or a reduction in lymphocytes will impair the functionality of lymphokine-activated killer cells and augment the propensity for distant metastases. Moreover, neutrophils participate in the induction of tumor suppressor gene mutations, the degradation of immunoglobulins, the degradation of receptors and complements, as well as the promotion of tumor cell proliferation and differentiation. Lymphocytes are the smallest category of leukocytes. Lymphocytes are seen as crucial in the innate immune defense against malignant tumors. The diminished lymphocyte count indicates a compromised function of CD8 cytotoxic lymphocytes in tumor cell eradication, resulting in a weakened T lymphocyte-mediated anti-tumor response and an overall loss in the body’s anti-tumor capacity. The reduction of lymphocyte count in the blood has been identified as an independent prognostic indicator for several malignancies [15].

The neutrophil-to-lymphocyte ratio (NLR) has been increasingly recognized as a valuable biomarker for cancer stratification, demonstrating significant associations with tumor burden, disease stage, metastatic potential, and lymphovascular invasion. In addition, NLR appears to exert a distinct prognostic influence on overall survival, disease-free survival, and cancer-specific survival [16].

Nevertheless, its predictive significance in EC has been insufficiently explored, and the relationship between NLR and clinical outcomes in this malignancy remains equivocal. This study aims to assess the prognostic significance of preoperative neutrophil-to-lymphocyte ratio (NLR) in patients undergoing primary surgical treatment for endometrial cancer, introducing a population-specific NLR cutoff derived via ROC analysis to enhance prognostic precision in early-stage disease of EC.

## 2. Materials and Methods

### 2.1. Study Design and Setting

This retrospective observational cohort study was conducted at the Oncology Institute “Prof. Dr. Ion Chiricuță” in Cluj-Napoca, Romania. Data were retrospectively collected for 398 patients who underwent surgical staging procedures for endometrial cancer between 1 January 2013 and 31 December 2017.

### 2.2. Patient Selection

Patients were eligible for inclusion if they met all of the following criteria: (1) total abdominal hysterectomy with or without lymphadenectomy; (2) complete blood count performed on the day prior to surgery; (3) pathological confirmation of endometrioid endometrial adenocarcinoma (EEA) or mixed-type adenocarcinoma with a predominant endometrioid component; and (4) FIGO surgical stage I or II, according to the 2009 FIGO staging system. Patients characteristics are shown in Table 1.

Exclusion criteria included other surgical interventions not involving total abdominal hysterectomy, absence of preoperative blood count data from the day prior to surgery, histologic subtypes other than adenocarcinoma, FIGO stage III or higher, other cancers, and hematological diseases; the presence of acute infection was inherently excluded through routine preoperative clinical assessment and institutional surgical protocols, while the use of steroids was not documented.

### 2.3. Data Collection

Clinical and laboratory data were retrieved from patients’ electronic medical records. Recorded variables included age at diagnosis, type of surgical intervention, and FIGO stage, as well as neutrophil (NEU) and lymphocyte (LYM) counts obtained from the complete blood count (CBC) routinely performed one day prior to surgery. The neutrophil-to-lymphocyte ratio (NLR) was defined and calculated as the absolute neutrophil count divided by the absolute lymphocyte count.

### 2.4. Surgical and Histopathological Evaluation

All patients underwent total abdominal hysterectomy with bilateral salpingo-oophorectomy (TH/BSO) as primary treatment. Depending on intraoperative findings and tumor extent, additional procedures included pelvic and/or para-aortic lymphadenectomy, omentectomy, parieto-colic biopsies, and omental biopsies. Surgical decisions were made at the discretion of the treating surgeon.

Histopathological examination of surgical specimens included assessment of tumor type, grade, lymphovascular space invasion (LVSI), and the number of lymph nodes excised. Patients were stratified according to the number of lymph nodes removed (0, 1–5, or >5). Tumor staging was performed based on the 2009 FIGO classification system.

### 2.5. Statistical Analysis

Data collection was conducted using the Microsoft Office 365 Suite—Office Excel. Data processing, statistical analysis, and chart generation were performed using IBM^®^ SPSS^®^ Statistics, version 26.0 (IBM Corporation, Armonk, NY, USA). Distribution analysis for continuous variables was assessed using the Kolmogorov–Smirnov and Shapiro–Wilk tests. Group comparisons were performed using the Mann–Whitney U test.

Univariate survival analysis was carried out using the Kaplan–Meier method with log-rank testing, as well as univariate Cox proportional hazards modeling. Multivariate survival analysis was conducted using multivariate Cox proportional hazards regression models.

All statistical tests were considered significant at a two-tailed *p*-value < 0.05. The outputs of the statistical analysis included *p*-values, group comparison charts, and residual plots.

## 3. Results

This retrospective analysis included a total 398 patients diagnosed with endometrial cancer, with a mean age of 60.56 years (SD = 9.11; 95% CI: 59.66–61.45) and a median age of 60 years. Histopathological examination revealed that 43.47% of tumors were well-differentiated (G1), 41.71% were moderately differentiated (G2), and 14.82% were poorly or undifferentiated (G3). Surgical staging according to the FIGO 2009 criteria identified 44.44% of patients in stage IA, 37.12% in stage IB, and 18.43% in stage II. The median number of lymph nodes excised was 2 (range: 0–68), with a mean of 6.22 nodes (SD = 10.73). Most patients (71.85%) had between 0 and 5 lymph nodes removed. Regarding nodal status, 27.39% of patients were pN0, 3.77% were pN1, 0.50% were pN2, and 68.34% were pNx. Lymphovascular space invasion (LVSI) was identified in 19.40% of cases, while vascular and perineural invasion were less frequent, reported in 1.51% and 0.25% of cases, respectively. Surgical margins were negative (R0) in 93.47% of patients, microscopically positive (R1) in 6.28%, and grossly positive (R2) in 0.25%.

These findings offer critical insight into the tumor characteristics and establish the basis for future prognostic and therapeutic evaluations in endometrial cancer.

Hematological parameters were analyzed preoperatively, with particular focus on neutrophil count, lymphocyte count, and the neutrophil-to-lymphocyte ratio (NLR). The mean neutrophil count was 5.05 × 10^3^/µL (SD = 2.78; 95% CI: 4.78–5.33), while the mean lymphocyte count was 2.11 × 10^3^/µL (SD = 0.91; 95% CI: 2.02–2.20). The mean NLR was 2.93 (SD = 3.47; 95% CI: 2.58–3.27), with a median of 2.21 and a range of 0.69 to 44.33. The distribution of NLR values was notably right-skewed (skewness = 7.95) and leptokurtic (kurtosis = 82.34), reflecting the presence of patients with markedly elevated systemic inflammatory response.

To define a clinically relevant threshold for the neutrophil-to-lymphocyte ratio (NLR), we performed Receiver Operating Characteristic (ROC) curve analysis using overall survival as the outcome. The optimal cutoff value of 2.27 was selected based on the coordinate point that maximized the Youden index, ensuring the best balance between sensitivity and specificity within our cohort. These findings support the utility of applying a predefined cut-off value—such as 2.27 derived from ROC analysis—for prognostic stratification in endometrial cancer.

By outlining key demographic and clinical variables, the descriptive statistics serve to contextualize subsequent analyses examining the prognostic significance of preoperative inflammatory markers, particularly NLR, in relation to oncologic outcomes.

Within the scope of this investigation, ROC curve analysis was utilized to assess the significance of clinical factors in terms of prognostic significance in patients diagnosed with endometrial cancer.

**Figure 1:**
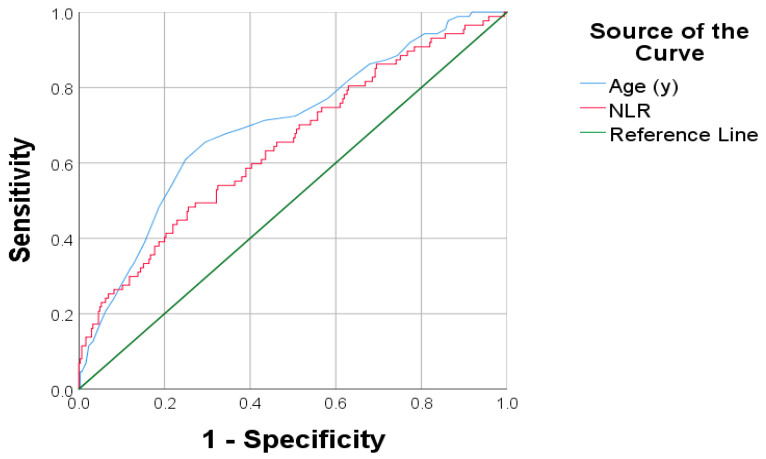
ROC curves for age and NLR as predictors of OS in patients with endometrial cancer.

The results demonstrated that both age and NLR are significant independent predictors of overall survival. An age threshold of ≥64 years yielded an area under the curve (AUC) of 0.699, indicating good discriminative ability, whereas an NLR cutoff of ≥2.27 was associated with an AUC of 0.646, suggesting moderate predictive capacity. Both factors achieved statistical significance (*p* < 0.001), supporting their roles as valuable independent prognostic markers in this patient cohort (Figure 1).

**Figure 2:**
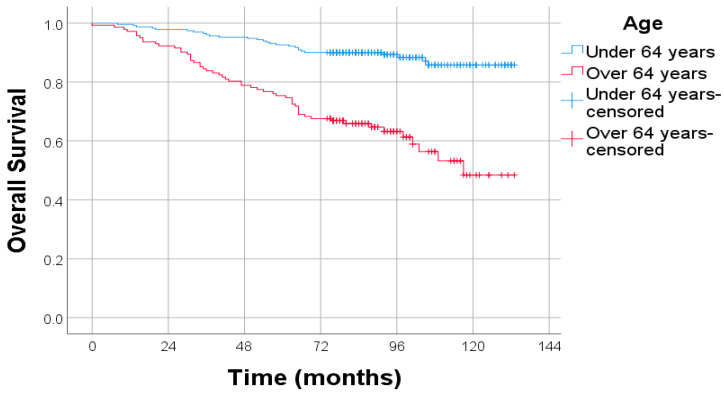
Kaplan–Meier survival curves for OS stratified by age. Log-rank test: *p* = 0.001; HR: 4.084; 95% CI: 2.572–6.485.

A statistically significant reduction in overall survival (OS) was observed among patients aged 64 years and above, as demonstrated by Kaplan–Meier analysis. Patients aged ≥ 64 years exhibited substantially reduced survival compared to those under 64 years. The separation between the survival curves was statistically significant, as confirmed by the log-rank test (*p* = 0.001), with a hazard ratio (HR) of 4.084 (95% CI: 2.572–6.485). These findings indicate that age is a strong and independent prognostic factor for OS in endometrial cancer patients (Figure 2).

**Figure 3:**
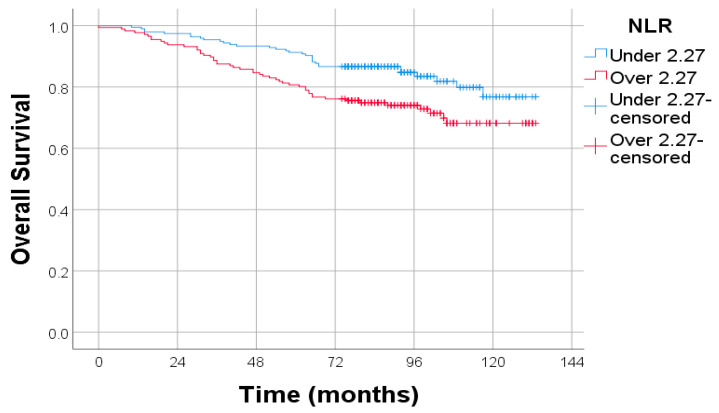
Kaplan–Meier survival curves for OS stratified by NLR. Log-rank test: *p* = 0.008; HR: 1.819; 95% CI: 1.164–2.839.

Kaplan–Meier survival analysis revealed a significant association between OS and preoperative NLR levels. Patients with an NLR ≥ 2.27 had markedly reduced OS compared to those with NLR < 2.27. This difference was statistically significant according to the log-rank test (*p* = 0.008), with a HR of 1.819 (95% CI: 1.164–2.839). These findings suggest that elevated NLR is an independent negative prognostic factor in endometrial cancer (Figure 3).

**Figure 4:**
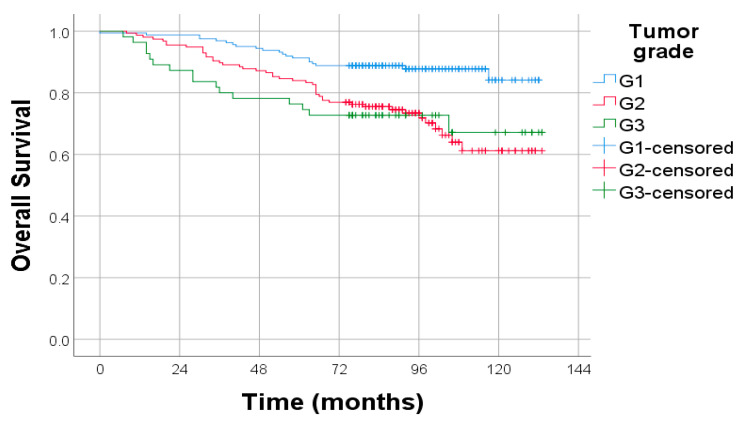
Kaplan–Meier survival curves for OS stratified by tumor histological grade. Log-rank test: *p* < 0.001.

Kaplan–Meier survival analysis demonstrated a significant association between tumor histological grade and OS. Patients with well-differentiated tumors (G1) had the most favorable survival outcomes, followed by those with moderately differentiated tumors (G2), while patients with poorly or undifferentiated tumors (G3) had the poorest OS. The difference between the survival curves was statistically significant (log-rank test, *p* < 0.001), indicating that histological grade is a strong prognostic factor for OS in endometrial cancer (Figure 4).

**Figure 5:**
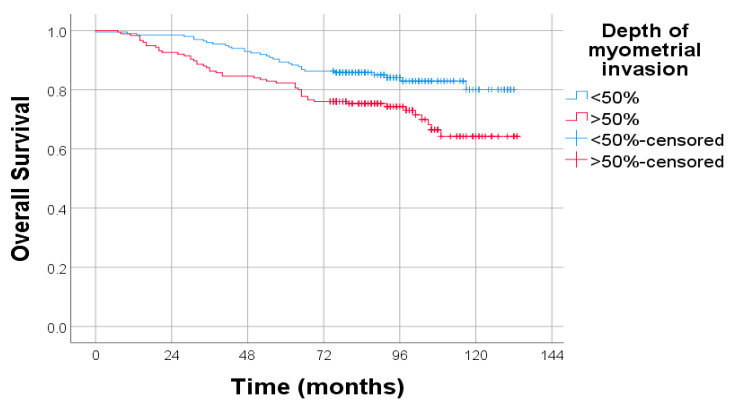
Kaplan–Meier survival curves for OS stratified by depth of myometrial invasion.

A clear disparity in OS was observed between patients with different depths of myometrial invasion. Those with >50% invasion had significantly poorer survival compared to patients with ≤50% invasion. This finding supports the prognostic relevance of deep myometrial infiltration in endometrial cancer. The difference between the survival curves was statistically significant (*p* = 0.04), indicating that depth of invasion is associated with long-term outcomes(Figure 5).

**Figure 6:**
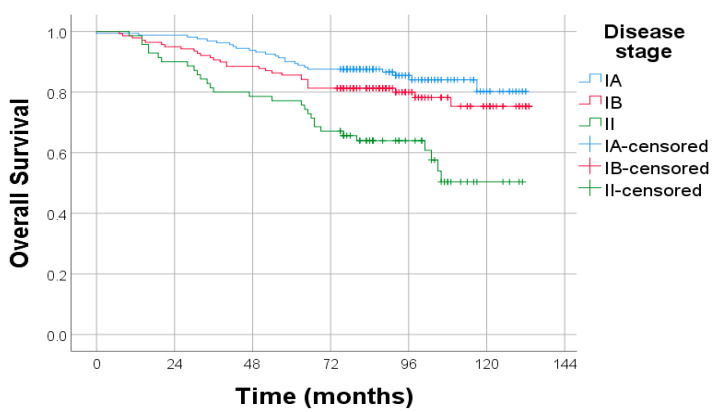
Kaplan–Meier survival curves illustrating OS according to FIGO 2009 surgical. Log-rank test: *p* < 0.001.

The extent of disease at diagnosis remained a significant determinant of survival. Patients with stage IA disease had the most favorable prognosis, while survival declined progressively in those with stage IB and was poorest in stage II. The difference in survival across these FIGO stages was statistically significant (*p* < 0.001), underscoring the prognostic relevance of early-stage detection and surgical staging in endometrial cancer management (Figure 6).

**Figure 7:**
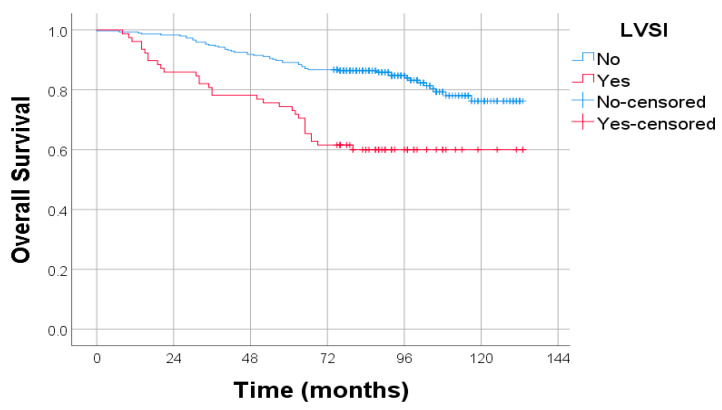
Kaplan–Meier survival curves for overall survival OS stratified by the presence or absence of LVSI. Log-rank test: *p* < 0.001.

The presence of lymphovascular space invasion (LVSI) was strongly associated with decreased overall survival (OS). Patients with LVSI experienced significantly poorer survival compared to those without, as evidenced by the clear separation of the Kaplan–Meier curves. This association was statistically significant (*p* < 0.001), highlighting LVSI as a key adverse prognostic factor in endometrial cancer (Figure 7).

**Figure 8:**
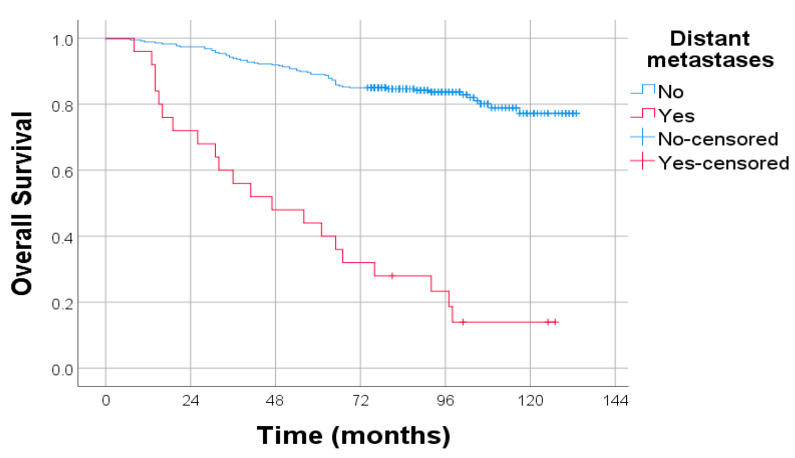
Kaplan–Meier survival curves for OS in patients with and without distant metastases. Log-rank test: *p* < 0.001.

Distant metastases were associated with a dramatic reduction in OS. As shown in the Kaplan–Meier curves, patients with metastatic disease had significantly poorer outcomes compared to those without, with a steep decline in survival over time. This difference was highly statistically significant (*p* < 0.001), underscoring distant metastasis as one of the most powerful predictors of mortality in endometrial cancer (Figure 8).

To assess the influence of resection margin status on the selection of adjuvant treatments, a series of Chi-square analyses were conducted across all therapeutic modalities administered postoperatively. Resection margins were categorized as R0 (negative), R1 (microscopically positive), or R2 (macroscopically positive).

**Figure 9:**
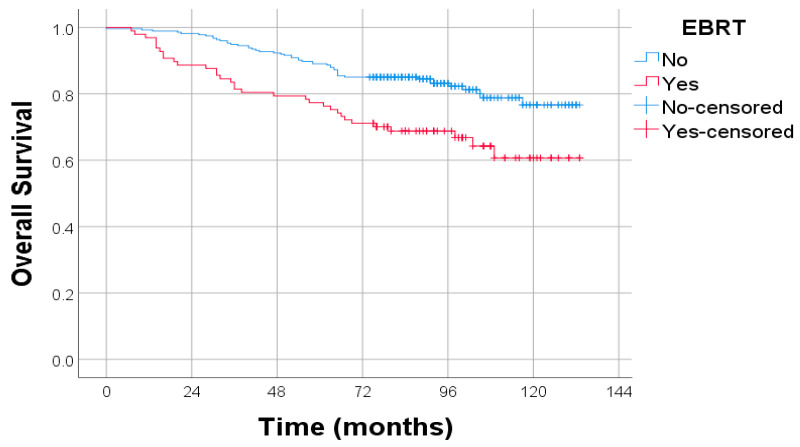
Kaplan–Meier survival curves for overall survival (OS) stratified by resection margin status. Censored observations are marked with “+” symbols. Statistical comparison by log-rank test: *p* = 0.006.

A statistically significant association was observed between margin status and the use of external beam radiotherapy (EBRT) (χ^2^ test, *p* = 0.004). Among patients with R1 margins, 52.0% received EBRT, compared to only 22.6% of patients with R0 margins. This finding supports the role of EBRT as a recommended intervention in the context of incomplete microscopic tumor excision, consistent with current clinical guidelines (Figure 9).

In contrast, no statistically significant associations were found between margin status and the administration of other adjuvant treatments. Specifically, the use of brachytherapy (*p* = 0.492), systemic chemotherapy (*p* = 0.848), hormonal therapy (*p* = 0.868), and radiosensitizing chemotherapy (*p* = 0.999) did not differ significantly based on margin involvement. These results suggest that the decision to initiate these treatments is influenced by additional clinical or pathological risk factors, rather than the resection status alone.

In addition to influencing treatment selection, resection margin status was also found to have a significant impact on OS. Kaplan–Meier survival analysis demonstrated a statistically significant difference in OS between margin groups (log-rank test, *p* = 0.006), with patients in the R1 category exhibiting inferior survival compared to those with R0 resections. This reinforces the prognostic relevance of complete tumor excision and highlights the importance of margin status not only in guiding adjuvant treatment decisions but also as an independent factor influencing patient outcomes.

**Figure 10:**
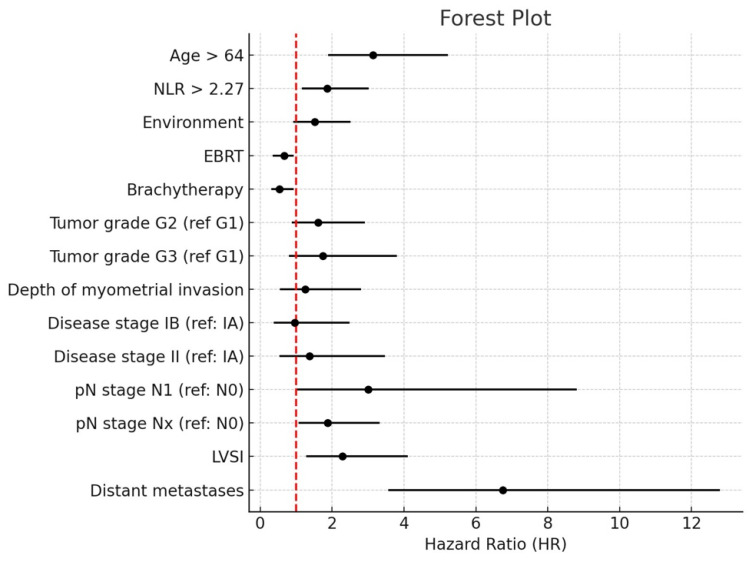
Forrest plot associated with multivariate Cox proportional hazards model based on the influence of various predictors on OS. The black dots represent the estimated hazard ratios (HR) and the horizontal lines indicate the 95% confidence intervals. The red dashed vertical line corresponds to HR = 1.

Multivariate Cox proportional hazards analysis identified several variables independently associated with OS in patients with endometrial cancer. Advanced age and an elevated NLR remained statistically significant predictors of inferior survival outcomes. Regarding adjuvant treatment modalities, vaginal brachytherapy was associated with a statistically significant reduction in the risk of death, suggesting a protective effect. In contrast, external beam radiotherapy (EBRT) and tumor grade did not demonstrate independent prognostic value in the adjusted model. The presence of LVSI was significantly correlated with reduced OS. Similarly, lymph node involvement—specifically pN stage 1 and pN stage 3—emerged as independent adverse prognostic factors. Among all parameters evaluated, the presence of distant metastases (M1) conferred the highest risk, thereby constituting the most powerful negative prognostic factor in the model (Figure 10).

**Figure 11:**
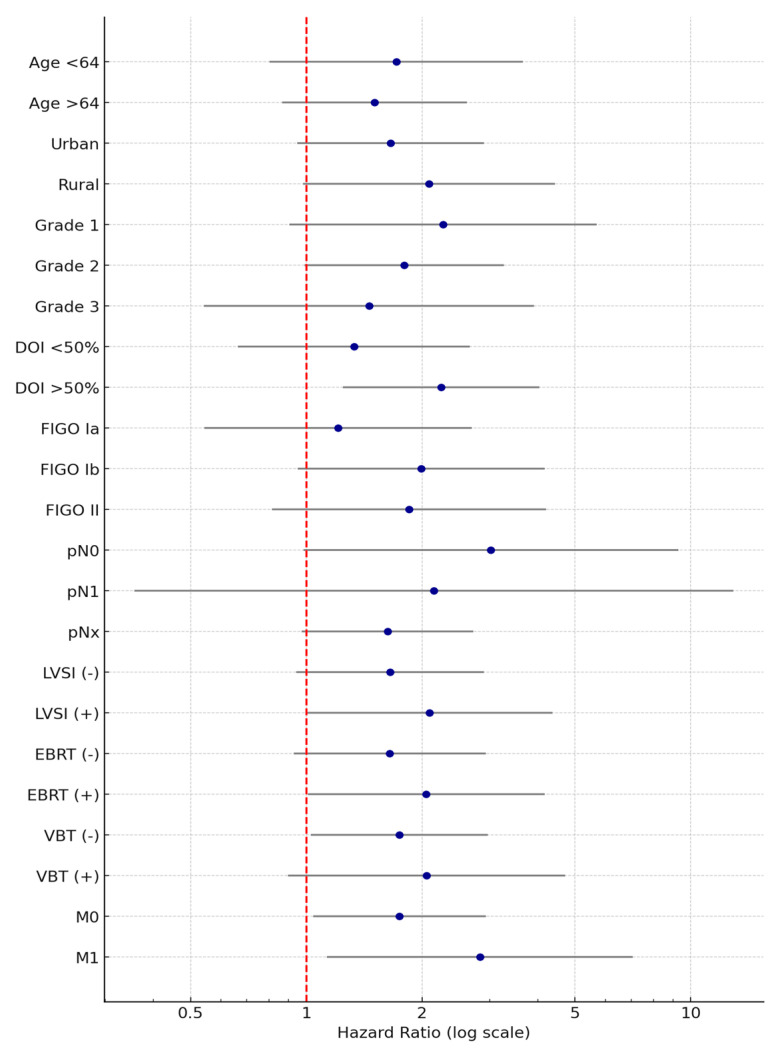
Forest plot illustrating hazard ratios (HRs) and 95% confidence intervals (CIs) derived from univariate Cox regression analysis for NLR influence (over vs. under 2.27) on overall survival (OS) in patients with endometrial cancer. Blue dots represent HR estimates with 95% CIs, and the red dashed line indicates HR = 1.

To investigate the independent prognostic value of the NLR on OS, a multivariate Cox proportional hazards model was constructed, incorporating a comprehensive panel of clinical, pathological, and treatment-related variables. The resulting forest plot presents the adjusted HR and corresponding 95% CI for each covariate included in the analysis. An elevated NLR (>2.27) remained an independent predictor of adverse overall survival, with a HR substantially exceeding 1 and a confidence interval that did not cross the null value, indicating statistical significance after adjustment for potential confounders (Figure 11).

Notably, advanced age (>64 years), presence of LVSI, and distant metastases (M1) were also independently associated with significantly reduced OS, with their hazard ratios indicating robust prognostic impact. In contrast, tumor differentiation grade, extent of myometrial invasion, FIGO stage, and adjuvant radiotherapeutic modalities did not retain statistical significance within the adjusted model, suggesting a more limited influence on long-term survival when considered concurrently with systemic inflammatory and metastatic parameters.

These findings reinforce the role of NLR as a potential accessible, cost-effective inflammatory biomarker with independent prognostic relevance in endometrial cancer and highlight its potential utility in preoperative risk stratification and personalized therapeutic decision-making.

## 4. Discussion

The findings of our study highlight the prognostic relevance of the preoperative NLR in patients with endometrial cancer undergoing primary surgical treatment. Our data demonstrate that an elevated NLR (≥2.27) is significantly associated with reduced OS, independent of other well-established prognostic factors such as age, FIGO stage, LVSI, depth of myometrial invasion, and the presence of distant metastases. The independent prognostic significance of NLR, with a HR of 1.87 (95% CI: 1.156–3.017; *p* = 0.011) in multivariate Cox regression, suggests its potential utility as a reliable biomarker for stratifying risk in this patient population. Previous research by Benedetti Panici et al. reported that age over 65 years is a strong independent prognostic factor, with a significant reduction in OS among patients older than 65 compared to those aged 65 or younger [17]. In our cohort, the cutoff value for age was 64 years, and its correlation with OS was confirmed by the Chi-square test, yielding a *p*-value of 0.001 and a HR of 4.084 (95% CI: 2.572–6.485). Therefore, we can conclude that in patients older than 64 years, overall survival is reduced by a factor of four compared to those younger than 64 years. Biologically, NLR is considered a surrogate marker of systemic inflammatory response, reflecting a relative increase in circulating neutrophils—which are known to promote tumor progression through angiogenesis, extracellular matrix remodeling, and immune evasion—and a decrease in lymphocytes, which play a central role in anti-tumor immune surveillance. Consequently, an elevated NLR may indicate a pro-tumoral immunological environment, potentially contributing to worse clinical outcomes. Our findings are consistent with several previously published studies. Dong et al. reported that a higher NLR (cutoff ≥ 2.47) was independently associated with worse OS, cancer-specific survival (CSS), and disease-free survival (DFS) in a cohort of 510 patients with surgically treated endometrial cancer [18]. Similarly, a comprehensive meta-analysis by Ni et al., which included data from 3390 patients across nine studies, found that elevated pretreatment NLR was significantly associated with poor OS (pooled HR = 2.22, 95% CI: 1.77–2.78) and PFS (HR = 1.81, 95% CI: 1.35–2.41), reinforcing the negative prognostic implications of systemic inflammation in endometrial cancer [19].

Li et al. further expanded on this concept by incorporating NLR into prognostic nomograms alongside fibrinogen, albumin, and CA125 levels. Their study involving 1483 patients demonstrated that NLR ≥ 2.521 was independently predictive of poor OS and PFS, and that nomograms constructed with these markers yielded high calibration and discrimination metrics, supporting their integration into clinical practice [20]. In the context of high-grade endometrial carcinomas, Zhang et al. developed a multifactorial prognostic model that included NLR and the PLR. This model demonstrated excellent predictive performance for OS and DFS, particularly in distinguishing high- and low-risk subgroups within a large multicenter cohort of 910 patients [21]. Subgroup analysis in our cohort revealed that the prognostic impact of elevated NLR was particularly pronounced among patients with adverse clinicopathological features, such as LVSI, deep myometrial invasion, and distant metastases. This observation suggests that systemic inflammation, as indexed by NLR, may exacerbate the biological aggressiveness of tumors, thereby compounding the negative effect of established high-risk features. Importantly, our findings enhance the utility of preoperative inflammatory markers as adjunctive risk stratification tools. As a low-cost, non-invasive, and easily reproducible parameter, NLR could be seamlessly incorporated into pre-treatment assessment protocols. This would allow clinicians to better stratify patients into risk categories, guide the use of adjuvant therapies, and inform prognostic counseling.

Emerging evidence, including studies by Holub et al. and Njoku et al. have shown that inflammatory indices such as the systemic immune-inflammation index (SII) and PLR also possess prognostic value in gynecologic malignancies, further underscoring the value of systemic inflammation markers in risk assessment [22,23].

Muzykiewicz et al. assessed the prognostic significance of NLR and PLR in patients diagnosed with advanced-stage endometrial cancer (FIGO III–IV). Results showed that higher NLR was significantly associated with worse overall survival and progression-free survival, independent of disease stage. This confirmed NLR’s value as an independent prognostic marker in advanced gynecologic malignancy [24].

Leng et al. confirms that pretreatment elevated neutrophil-to-lymphocyte and platelet-to-lymphocyte ratios are robust biomarkers for poor prognosis in endometrial cancer, particularly affecting overall and disease-free survival. These markers likely reflect an imbalance in the host immune response and tumor-promoting inflammation, which have been implicated in cancer progression and resistance to therapy [25].

Our study reinforces the prognostic significance of the neutrophil-to-lymphocyte ratio (NLR) in endometrial cancer. Using preoperative complete blood counts, we identified an optimal NLR cutoff of 2.27 via ROC analysis. This threshold was significantly associated with survival outcomes, as demonstrated through both Kaplan–Meier survival curves and Cox proportional hazards modeling. Reported NLR cutoff values in endometrial cancer studies vary considerably, often ranging from 2.0 to 5.0, depending on population characteristics and statistical methodology. Our analysis used ROC curve methodology to derive a cohort-specific cutoff (2.27), tailored to the biological and clinical features of early-stage endometrial cancer patients treated surgically. This data-driven approach enhances prognostic accuracy and supports the development of more refined risk stratification tools. In this setting our findings suggest that elevated NLR is independently predictive of poorer prognosis, aligning with the growing recognition of inflammation-based markers in oncologic risk stratification.

These results are consistent with the earlier findings of Haruma et al., who evaluated a Japanese cohort of 320 endometrial cancer patients and reported NLR cutoffs of 2.41 for PFS and 2.70 for OS. Their study similarly found that elevated pre-treatment NLR values were significantly correlated with shortened DFS and OS. The proximity of our identified cutoff (2.27) to those reported by Haruma et al. strengthens the external validity of NLR as a clinically relevant prognostic biomarker [26]. Notably, while slight variations in optimal cutoff values may reflect population differences, cancer staging, or methodological choices in ROC analysis, both studies underscore a consistent relationship between systemic inflammation—as quantified by NLR—and adverse outcomes in endometrial cancer.

Given its accessibility and low cost, the CBC is one of the most requested baseline investigations during initial patient evaluations. Its cost-effectiveness stems not only from its affordability but also from its widespread availability, as nearly all healthcare facilities involved in this study routinely offer this laboratory test. Additionally, although CBC is a routine investigation, it enables the derivation of the NLR, offering additional prognostic insight without necessitating further testing [27]. NLR could serve as a valuable addition to existing preoperative risk assessment protocols, helping to guide clinical decision-making and patient counseling.

Cupp et al. provided a pivotal contribution through their comprehensive umbrella review, which systematically synthesized data from numerous cancer types and confirmed the consistent association between elevated NLR and adverse survival outcomes. Notably, their work highlights that elevated NLR correlates not only with overall survival but also with disease-free survival, suggesting that this hematological parameter captures underlying tumor biology reflective of a pro-inflammatory, immunosuppressive microenvironment conducive to tumor progression [28].

Although the present study does not specifically address recurrent endometrial cancer or immunotherapy, emerging evidence suggests that systemic inflammatory markers such as the neutrophil-to-lymphocyte ratio (NLR) may hold significant clinical value in these contexts. Barrington et al. demonstrated that in patients with recurrent endometrial cancer treated with immunotherapy, a lower baseline NLR (<6) was associated with substantially improved overall survival, with median survival nearly doubling compared to those with elevated NLR [29]. This highlights the broader prognostic and potentially predictive utility of NLR beyond its established role in primary disease settings.

Compared to, our study introduces key methodological and clinical advancements. With a large, homogeneous cohort (n = 398) of early-stage endometrial adenocarcinoma, we applied ROC analysis to define a population-specific NLR cutoff (2.27), improving precision over studies using generalized thresholds. Multivariate analysis confirmed NLR ≥ 2.27 as an independent predictor of overall survival, alongside classical factors such as age, LVSI, and nodal status. The size and uniformity of our cohort reinforce the prognostic relevance of NLR, strengthening its validity across early-stage disease. Moreover, our model integrated detailed surgical and treatment variables, rarely accounted for in earlier work. These features highlight the added prognostic value of NLR and support its incorporation into individualized preoperative risk stratification.

From a clinical perspective, the integration of NLR into routine preoperative assessment could offer a simple and cost-effective means of enhancing prognostic stratification in endometrial cancer. Given that complete blood counts are already standard in surgical oncology workflows, calculating NLR would require no additional testing or infrastructure. Patients identified as high-risk based on elevated NLR could benefit from closer postoperative monitoring, consideration for adjuvant therapy, or enrollment in clinical trials targeting immune or inflammatory pathways. However, before such integration, prospective studies are essential to validate its prognostic accuracy and define standardized thresholds across diverse populations.

This study is subject to several limitations. Its retrospective design inherently limits control over confounding variables and may introduce selection bias. The single-centre setting may restrict the generalizability of findings to broader patient populations. Additionally, some clinical parameters were incompletely or inconsistently documented. Notably, corticosteroid use—which can significantly affect systemic inflammatory markers such as NLR—was not systematically recorded. Similarly, comorbid conditions were not comprehensively assessed, limiting their inclusion in multivariate modeling and potentially introducing unmeasured confounding. Finally, the absence of molecular subtypes, precludes integration of NLR findings within established genomic prognostic frameworks.

## 5. Conclusions

This study confirms that the preoperative neutrophil-to-lymphocyte ratio (NLR) is a statistically significant and independently predictive biomarker of overall survival in patients with endometrial cancer. In a large, homogeneous cohort, we identified an optimal cutoff value (NLR ≥ 2.27), which was associated with nearly a twofold increased risk of mortality, independent of established clinical and pathological factors.

Given its accessibility, low cost, and ease of measurement, NLR offers a practical tool for enhancing preoperative risk stratification. Biologically, its prognostic value reflects the interplay between tumor-promoting inflammation and impaired host immunity. These findings support the incorporation of NLR into individualized treatment planning and risk-adapted management strategies. However, given its non-specific nature and the retrospective design of our study, prospective validation in multicenter settings is warranted to confirm clinical utility and refine integration with molecular classifiers. Until then, NLR should be viewed as a promising but adjunctive biomarker, to be interpreted alongside conventional prognostic indicators.

## Figures and Tables

**Table 1:** Overview of demographic, surgical, histopathological, and treatment characteristics of the study population.

Variable	Absolute Value (Number)	Percentage (%)
**Total number of patients**	398	
**Median age (years)**	60	
Age 29–39	10	2.51%
Age 40–49	34	8.54%
Age 50–59	133	33.42%
Age 60–69	153	38.44%
Age 70–79	65	16.33%
Age 80–89	3	0.75%
**Demographics**		
Urban background	267	67.09%
Rural background	131	32.91%
**Surgical details**		
Lymphadenectomy	132	33.17%
Omentectomy	35	8.79%
Parieto-colic biopsy	11	2.76%
Epiploic biopsy	13	3.27%
**Number of removed lymph nodes**		
Number of lymph nodes removed: 0	154	38.69%
Number of lymph nodes removed: 1–5	132	33.17%
Number of lymph nodes removed: >5	112	28.14%
**Resection margins**		
Resection margins R0	372	93.47%
Resection margins R1	25	6.28%
Resection margins R2	1	0.25%
**Pathological status of lymphnodes**		
Lymph node stage (pN): N0	109	27.39%
Lymph node stage (pN): N1	15	3.77%
Lymph node stage (pN): N2	2	0.50%
Lymph node stage (pN): Nx	272	68.34%
**FIGO staging**		
FIGO 2009 stage IA	177	44.44%
FIGO 2009 stage IB	148	37.12%
FIGO 2009 stage II	73	18.43%
**Tumor grade**		
Tumor grade G1	173	43.47%
Tumor grade G2	166	41.71%
Tumor grade G3	59	14.82%
**Lymphatic, vascular and perineural invasion**		
Lymphovascular invasion (L1)	77	19.40%
Vascular invasion (V1)	6	1.51%
Perineural invasion (Pn1)	1	0.25%
**Inflammatory NLR marker**		
Preoperative NLR: 0–2	166	41.71%
Preoperative NLR: 2–4	180	45.23%
Preoperative NLR: >4	52	13.06%
**Treatment decision**		
Therapeutic decision: surveillance	189	47.48%
Therapeutic decision: adjuvant treatment	146	36.68%
Therapeutic decision: unavailable	63	15.82%
**Treatment modalities**		
Brachytherapy	108	27.14%
External beam radiotherapy (EBRT)	98	24.62%
Adjuvant chemotherapy	37	9.29%
Concurrent chemoradiation	6	1.50%
Hormonal therapy	4	1.00%
**Treatment parameters**		
EBRT dose: 50Gy/25 fractions	60	63.16%
EBRT dose: 46Gy/23 fractions	22	23.16%
EBRT dose: other protocols	13	13.68%
BT dose: 6Gy × 2 fractions	33	30.56%
BT dose: 5Gy × 5 fractions	29	26.85%
BT dose: other protocols	46	42.59%
Number of adjuvant chemotherapy cycles: 6	20	54.05%
Number of adjuvant chemotherapy cycles: 4	8	21.62%
**Outcomes**		
Distant metastases	34	8.54%
Death	88	22.11%
Local recurrence	13	3.27%

NLR: Neutrophil-to-lymphocyte ratio, calculated from preoperative blood tests, EBRT: External beam radiotherapy. BT: Brachytherapy, pN staging and FIGO staging are based on surgical pathology, R0: clear margins; R1: microscopic residual disease; R2: macroscopic residual disease.

## Data Availability

The data presented in this study are available on request from the corresponding author. The data are not publicly available due to patient privacy.
